# Indents

**Published:** 1906-12-15

**Authors:** 


### INDENTS.

**☞ Brief Articles of Technical or Scientific Value will receive notice and comment. Contributions invited.**

**Humanitarian Dentistry.** Humanitarian Dentistry in the narrowest sense means that branch of dental practice which is devoted to the preventing and elimination of pain and suffering at the time the opertations are being performed. In the broader sense, the sense intended when the name was given, it means vastly more; it means that not only the comfort of the patient, but the comfort of the dentist must be looked after as well.—Ed. *Dentist Magazine*.

**Preparation of Cavities with Nitrous Oxid.** The preparation of cavities with nitrous oxide and oxygen is one of the most satisfactory methods I have ever used.

With this method I apply my rubber dam and prepare my cavity as far as possible, and then apply the nose piece and hold the patient with the nitrous oxide and oxygen until the cavity is completed. I have held a patient long enough to prepare two compound cavities in bicuspids. The draw back to this method is that the majority of patients do not like to submit to being anesthetized, and it is almost necessary no have a competent assistant to look after the anesthetic.—D. H. Ziegler, D.D.S., Cleveland, O.

**A New Solvent for Gold.** In a note recently published in the *Proceedings of the Chemical Society* on March 30, 1906, Dr. J. Moir points out that gold-leaf when floated on an acid solution of thiocarbamide readily passes into solution, the action being rapid when an oxidizing agent is added. Thus the gold-leaf dissolves after less than a minutes shaking, when a little ferric chloride, potassium dichromate, or hydrogen peroxide is introduced into the acidified thiocarbamide solution. The gold has been isolated from this solution in the form of a compound containing 45.42 per cent. of the metal. This compound may also be prepared by boiling sodium aurichloride with a solution of thiocarbamide.

**Silver Nitrate with Cement Fillings.** A cement filling, zinc oxyphosphate, placed upon a surface treated with silver nitrate, will for some reason, last a great deal longer and will be a great deal better mass than the same mass not having the peculiar effect it gets from this film of silver albuminate. A great many, no doubt, have seen the effect of silver nitrate upon a surface which has been infected to a very slight depth. That in time will become a polished black surface and further decay will never result. Now, if in deeper cavities we can make partial preparation and apply silver nitrate and have it last longer than otherwise, it is worth knowing—W.V. B. Ames, *Dental Review.*

**Be a Roycrofter.** We may not all agree with Elbert Hubbard in all his philosophy and theology, but all good dentist can heartily agree with him in one thing, that everything we do should be done the best we know how.

To do this entitles a man to the name of roycrofter—royal crafter of workman; that is fit to work for the king. I would that dentists, as much as any other workers on earth have the opportunity to taste the joy of doing fine work, to feel the satisfaction that is greater pay than dollars and cents for doing something artistic as well as useful. So get in line do everything the best you know, and be kind while doing it. Dr. W. J. Brady, *in Western Journal.*

**Rule for Topical Applications.** All topical remedies should be applied warm to sensitive cavities. Alcohol should be warmed before applying, and after this application, every effort should be devoted to prevent evaporation taking place rapidly enough to cause pain. The dentist who uses Alcohol, Chloroform, Ethyl Chloride, or Ether in a sensitive cavity, to produce a condition which will permit cutting without causing pain lacks all conception of what is required of him. He is a failure because he lacks judgmeat, (I am speaking now from the standpoint of Humanitarian Dentistry). He is absolutely worthless as a worker in this field, unless he can be taught the folly of trying to kill one pain by first causing a greater one— —.W. G. Ebersole in *Dentists Magazine*.

**Familiarity with Patients.** I would warn you against making an intimate friend of every fellow who happens to come into your practice. Now, I know a great many men who have a great deal of trouble with their practices.

They never get the fees they should receive and they are never paid as promptly as they ought to be, merely because they have established a certain degree of free and easy familiarity with every patient that comes under their care.

It is always these patients that are the first to impose on you in every way that a patient can impose on an operator, and so, while there should be a certain degree of affability displayed by the dentist, I think it should be associated with a professional dignity which should always be present in your relations with a patient and which will prevent them from attempting these little impositions that annoy, and that are expensive.—J. E. Nyman, *in Western Journal.*

**Local Anesthetic.** The following is my solution for the painless extration of teeth.

| | | |
|---|---|---|
| Cocaine hyd. | grs. | x |
| Carbolic Acid (cryst) | | |
| Gum Camphor | | |
| Chloral hydrate a. a. | grs. | III |
| Aqua dist., q. s. | ℥ | IV |

Subscription—Rub camphor and chloral together until liquified; add acid, cocaine and, lastly, water and filter.

Sig.—For painless extraction.

I have had it compounded a great many times at

25 cents. Compare the price with your $1.00 an ounce solution and compare it in any other way, for it will bear comparison. I compound my own solution and thus vouch for its accuracy. It takes effect immediately and I have my first case to hear from where sloughing followed its administrtion.—H. E. Dais, St. Joe, Ills., *Dental Review*.

**Treatment of Erupting Teeth.** I am convinced that most of the earache in children is due to the irritation of the erupting teeth, and at the same time suggest the use of antikamina as a panacea, and also the use of the lance in cases of convulsions where it is indicated, as I believe we are justified in using it when teeth do not show at the proper time. Even if it does not afford permanent relief, it does no harm, because supposing the gum heals and a cicatrix forms, it being of the lower form of tissue is more easily broken down by advancing teeth. Having seen many cases where the lance has been beneficial, I firmily believe in its intelligent use. In at least one case to which I was called the child was on the verge of convulsions, rolling the head and eyes, and in a state of evident stupor. The free use of the lance acted like magic, and in less than half an hour the little patient was enjoying a sound and natural sleep, the first in two or three days.—W. A. Robertson, *in Review.*

**Extraction of Molar Restores Sanity.** Dr. F. W. McCall, official dentist for the New York State Hospital, was called to that institution last week to extract an annoying tooth from the attractive face of a youthful female patient.

The girl was violently insane and had been in a deplorable mental condition for some time. She had just had some sort of a fit, and the physicians at the "Dippy College" decided that the best time to extract the tooth would be while she was still in a semi-conscious state. The dentist was accordingly sent for. The young lady had been in the habit of easing the pain of the tooth by biting the other inmates and attendants with it, so the doctor placed a wooden plug in her mouth and commenced operations. When the tooth was finally brought out to the light of day the patient gave an unearthly scream and was immediately restored to her right mind. She remained perfectly sane for some time and hopes are now entertained for her recovery.—*Binghampton Herald.*

**Fees.** Frequently you are asked for estimates as to the probable fee charged for a given operation. It has been my almost invariable practice for some time past to refuse to give any estimate at all, or when I do, I tell the patient it is merely an opinion and that I know very little more about what the proper charge for the operation will be than they do themselves. But I tell them, if they regard me as a conscientious, capable operator, and that I can and will perform the operation with judgment, honesty and skill, as it should be performed, they must also give me credit for being an honest commercial man who will charge only such a fee as is commensurate with the service rendered and which they can afford to pay. Tell them that never yet have you forced a patient to pay a bill which they really could not afford to pay, and which was in unjust proportion to their means. And we should be honest with patients in that respect; we should show charity toward people who are deserving of charity and we will not lose anything by it.—J. E. Nyman.

**Antrum Infected by the Teeth.** Dr. Curtis kindly suggested that at some future time I should present a paper bearing strictly upon the subject of the diseased antrum as caused by the teeth, and show more cases than I have done to-night. In answer to this I would say that in all my dissections of over a thousand skulls I have not found more than a dozen cases where I could say that the antrum had become diseased from the teeth. Those who practice oral surgery might naturally conclude that there are a great many such cases, because most of them are referred to the oral surgeon by dentists, physicians and rhinologists; my early experience leads me to believe such to be the case, but after a more careful study of the skull, particularly of the pneumatic spaces, it was found that these regions included such a large area of mucus-covered air spaces and that they are divided into so many sinuses, cells recesses and pockets, that they are liable to become diseased from infection which passes from one air space to another and as the maxillary sinus is the lowest of these pneumatic spaces, with its outlet at the top, it is more than likely to become diseased from infection and deposits from the spaces that are above. The nerves and blood vessels that supply the teeth pass along the floor of the maxillary sinus not within the bone as described in many of our text books on anatomy. This being the case the nourishment of the teeth is impaired and the teeth become diseased, so that I believe there are many more teeth lost through infection from the pneumatic spaces than there are sinuses infected from teeth.—M. H. Cryer *in Items.*

**Failures.** Now, then, as to your failures, because you will have them, as well as myself, and everyone else. There probably never was a failure that did not stagger a man a little bit. There are some men who allow failures to check their careers almost altogether. There is no reason why that should be. There is a value attached to every failure, paradoxical as that may seem. There is a lesson to be gained from every failure, and that is why it is of value to you.

When a bridge or crown comes back to you when it has failed, do not tear it out in absolute disgust and throw it in the scrap heap, but lay it to one side and after while when you have time, in a calmer mood, carefully study that thing and find out just where your mistake was, in that way you can insure not making it again.

Then have the courage to admit to your patient you have made a failure, when you really have made one. One of the most profitable admissions I ever made in my whole practice was when I told a certain gentlemen I had made a failure in an operation, and was going to try to find out what was lacking in it, so that I should not fail again. That one man's regard has done more to build up my practice than any other influence that I can recall, and it was I believe because he admired as he said, the courage and honesty that I showed in admitting that that failure was a failure, instead of trying to gloss it over and call it something else. You will lose nothing in the estimation of your patient when you frankly admit a failure is a failure, and assert that you are going to correct it and see that it does not occur again.—J. E. Nyman *in Western Journal.*

